# Use of sodium–glucose cotransporter‐2 inhibitors among Aboriginal people with type 2 diabetes in remote Northern Territory: 2012 to 2020

**DOI:** 10.1111/imj.16653

**Published:** 2025-02-24

**Authors:** Matthew J. L. Hare, Winnie Chen, Thomas Berhane, Sumaria M. Corpus, Louise J. Maple‐Brown

**Affiliations:** ^1^ Endocrinology Department Royal Darwin Hospital Darwin Northern Territory Australia; ^2^ Wellbeing and Chronic Preventable Diseases Division, Menzies School of Health Research Charles Darwin University Darwin Northern Territory Australia; ^3^ Danila Dilba Health Service Darwin Northern Territory Australia; ^4^ Division of Medicine Royal Darwin Hospital Darwin Northern Territory Australia; ^5^ Aboriginal & Torres Strait Islander Advisory Group, Diabetes across the Lifecourse: Northern Australia Partnership Menzies School of Health Research Darwin Northern Territory Australia

**Keywords:** indigenous health, type 2 diabetes, primary care, general practice, pharmacoepidemiology, rural health service

## Abstract

Aboriginal people in remote Northern Territory communities experience the highest burden of type 2 diabetes (T2D) globally. Sodium–glucose cotransporter‐2 inhibitors (SGLT2i) improve cardiac and renal outcomes in selected populations. However, safety in this context is unknown. We investigated SGLT2i use and outcomes in remote Aboriginal people with T2D between 2012 and 2020.

Aboriginal people living in remote areas of the Northern Territory (NT) experience some of the highest burden of type 2 diabetes (T2D) in the world, with a reported adult prevalence of diabetes of up to 40% in some areas of the NT.^1^ Diabetes in the NT is associated with high rates of complications, including chronic kidney disease (CKD) and cardiovascular disease.^2^, ^3^

Sodium–glucose cotransporter‐2 inhibitors (SGLT2i) improve cardiac and renal outcomes for select patient populations.^4^, ^5^ However, the safety profile of SGLT2i among Aboriginal people in the remote NT is unknown. Potential adverse effects include volume depletion, diabetic ketoacidosis (DKA), genitourinary infections and amputations. Heightened risk of such events is foreseeable in the NT due to climate, food insecurity, complex diabetes phenotypes^6^ and high background rates of infection^7^ and amputation.^8^ Thus, our study aimed to investigate the use and outcomes of SGLT2i for T2D in remote Aboriginal people living in NT between 2012 and 2020.

We investigated a retrospective cohort of all Aboriginal patients with T2D prescribed an SGLT2i at all 48 NT Health remote primary healthcare clinics in the NT, between October 2012 (first SGLT2i approved by Therapeutic Goods Administration) and June 2020. A map of included clinic sites is displayed in Figure 1. These clinics are all sole providers in remote Aboriginal communities. The included cohort was formed using extraction of prescription data from the Primary Care Information System (PCIS), which is the electronic medical record system used in all NT Health remote community clinics. No specific inclusion criteria were used to select patients with T2D; however, during the study period up to 2020, SGLT2i were only Pharmaceutical Benefits Scheme (PBS)‐indicated for T2D.^9^ Clinical data were collected via manual review of individual patient records in PCIS and also the electronic medical records system for all public hospitals in the NT. Baseline data collected included demographics, anthropometry, comorbidities, smoking status and medications. Clinical effectiveness indicators were compared between baseline, and at 6‐month intervals up to 2 years from SGLT2i commencement – these clinical indicators included haemoglobin A1c (HbA1c), weight, blood pressure, estimated glomerular filtration rate (eGFR) and urine albumin–creatinine ratio (uACR). Given the longitudinal nature of routinely collected data and possibility of multiple measurements, the closest reading (e.g. HbA1c) within 6 months of the relevant 6‐, 12‐, 18‐ and 24‐month interval was selected. Adverse events and discontinuation could occur at any time point while on SGLT2i therapy. Therefore, adverse events and discontinuation data were collected up until the end of the data collection period at January 2021. The NT Human Research Ethics Committee (NT HREC 2020‐3721), and Aboriginal and Torres Strait Islander Advisory Group of the Diabetes across the Lifecourse: Northern Australia Partnership approved the study.

**Figure 1:**
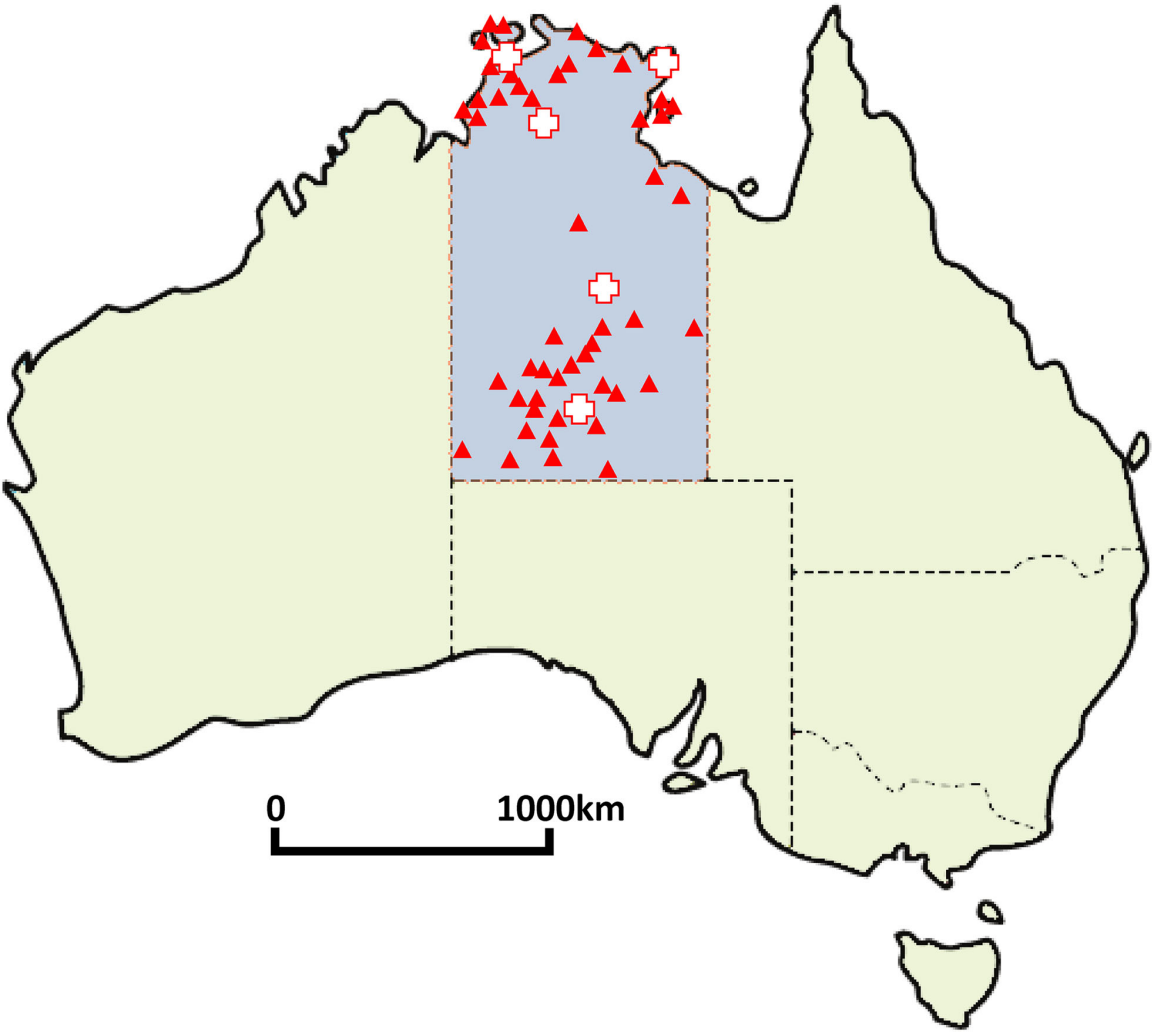
Map of included remote Aboriginal community sites in the Northern Territory. Red triangles represent each of the remote Aboriginal community sites included in this study. White crosses represent the location of the Northern Territory public hospitals.

A total of 519 Aboriginal people from 48 remote NT communities was included. The mean age of the cohort was 47 years (standard deviation 12 years), and 55% were female. There was a high proportion of people with comorbidities including 401 (77%) with CKD and 95 (18%) with cardiovascular disease (Table 1). The majority of people taking an SGLT2i were prescribed empagliflozin, and the number of new prescriptions during the study period increased over time (Fig. 2).

**Table 1:** Baseline characteristics

Age (years)	47 ± 12
Female	281 (55%)
Follow‐up (years)	2.0 (1.3–3.1)
HbA1c (%)†	9.9 ± 2.1
BMI (kg/m^2^)†	29.5 ± 6.7
Established CVD	94 (18%)
Hypertension	291 (57%)
Dyslipidaemia	439 (86%)
Current smoking	189 (37%)
CKD stage	
None	114 (22%)
Stage 1	217 (43%)
Stage 2	133 (26%)
Stage 3A	26 (5.1%)
Stage 3B	6 (1.2%)
Stage 4	2 (0.4%)
Stage unknown	12 (2.4%)

† Data available for *n* = 499 for HbA1c, *n* = 495 for BMI. Continuous variables are expressed in mean ± SD, categorical variables are expressed in percentages.

BMI, body mass index; CKD, chronic kidney disease; CVD, cardiovascular disease; HbA1c, haemoglobin A1c; SD, standard deviation.

**Figure 2:**
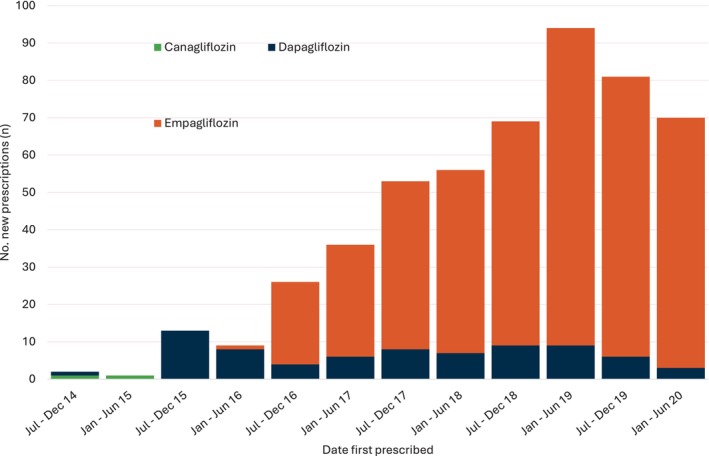
New sodium–glucose cotransporter‐2 inhibitors prescriptions per 6‐month period, 2012–2020.

At a median follow‐up of 2.0 years, use of SGLT2i was associated with modest improvements in HbA1c and weight (Table 2). There was minimal decline in renal function (eGFR, uACR) between baseline and follow‐up. At the end of the follow‐up period, 28% of patients discontinued therapy. Seventeen (3.3%) deaths, nine non‐traumatic amputations (1.8%), six severe hypoglycaemia events (1.2%), one DKA event (0.2%) and no cases of Fournier gangrene (0%) were observed.

**Table 2:** Comparison of clinical outcomes at baseline versus at the end of the follow‐up period

	*n*	Baseline	Follow‐up	*P*‐value
HbA1c (%)	457	9.9 ± 2.1	9.5 ± 2.3	0.002
Weight (kg)	410	87 ± 21	85 ± 21	<0.001
eGFR ≥ 90 mL/min/1.73 m^2^ (%)	429	279 (65%)	243 (57%)	<0.001
Urine ACR (mg/mmol)	395	12 (2.4–56)	13 (2.2–68)	0.011
Systolic BP (mmHg)	467	128 ± 18	127 ± 19	0.473

Distribution of urine ACR was skewed and thus reported as a median. Proportion of eGFR ≥90 was reported – noting that median eGFR was 90 for both the baseline and follow‐up groups. Paired *t* test was used for HbA1c, weight and systolic blood pressure. Wilcoxon signed rank test and McNemar chi‐squared tests were used for categorical variables.

ACR, albumin–creatinine ratio; BP, blood pressure; eGFR, estimated glomerular filtration rate; HbA1c, haemoglobin A1c.

## Discussion

We report the first study to our knowledge of SGLT2i use and outcomes among Aboriginal people in remote Australian communities. Australians with diabetes living in more remote and/or disadvantaged socioeconomic areas may have reduced access to SGLT2i and other newer diabetes medications,^10^ but this association has not been confirmed in Aboriginal and Torres Strait Islander populations.^11^ Our study is timely as expanded clinical indications for SGLT2i, including heart failure and CKD, are driving further increases in utilisation.^9^ The strength of our study is that it includes individual patient data from a large number of remote primary healthcare clinics. The NT‐wide cohort in this study has similar characteristics in terms of age and sex distribution but higher mean HbA1c (9.9% in this study, vs 7.9%) compared to our previous study of all patients with diabetes within the same remote Australian communities.^1^

In summary, we found that SGLT2i were being prescribed to people likely to benefit based on international trial evidence. The cohort being prescribed SGLT2i had a high prevalence of CKD, CVD and cardiovascular risk factors. SGLT2i prescription was accompanied by modest improvements in weight and HbA1c over time (reduction of 2 kg and 0.4% respectively), consistent with 2‐year findings of major international studies such as the EMPA‐REG and DECLARE trials^12^, ^13^ and a previous study within an urban Aboriginal and Torres Strait Islander cohort.^14^ Adverse events such as amputations and DKA (1.8% and 0.2% respectively) from this study are also comparable with other major trials.^15^

A limitation of this study was that only prescription data from the included electronic medical record system was available for analysis. Secondary use of electronic medical record data is subject to variations in data accuracy and completeness. Furthermore, routinely collected electronic medical record data do not provide information on medication dispensing or adherence, which has previously been described as a potential issue in remote primary NT Health remote primary health clinics.^16^ If medication adherence is optimised, it is plausible that the observed clinical effect of SGLT2i may be greater than what has been reported in this study.

While the findings of this study are largely reassuring, the possibility of higher rates of rare, but serious, adverse events in this unique context is not excluded given the limited sample size and lack of a comparison group. Future studies investigating the effectiveness and safety profile of SGLT2i in remote NT Aboriginal populations including a larger patient cohort, with matched controls, would be crucial as use of SGLT2i increases within these contexts.

## Data Availability

NT Health owns the data used for this research. Access to the study database would be subject to approval from the data owner as well as relevant ethics and Aboriginal and Torres Strait Islander research governance processes and principles.
